# Biogenic ZnO Nanoparticles Synthesized by *B. licheniformis*: A Selective Cytotoxicity Against NG-108 Glioblastoma Cells

**DOI:** 10.3390/nano15171338

**Published:** 2025-08-31

**Authors:** Alberto Bacilio Quispe Cohaila, Gabriela de Lourdes Fora Quispe, César Julio Cáceda Quiroz, Roxana Mamani Anccasi, Telmo Agustín Mejía García, Rocío María Tamayo Calderón, Francisco Gamarra Gómez, Elisban Juani Sacari Sacari

**Affiliations:** 1 Grupo de Investigación GIMAECC, Facultad de Ingeniería, Universidad Nacional Jorge Basadre Grohmann, Avenida Miraflores S/N, Ciudad Universitaria, Tacna 23003, Peru; 2 Escuela de Metalurgia y Materiales, Facultad de Ingeniería, Universidad Nacional Jorge Basadre Grohmann, Avenida Miraflores S/N, Ciudad Universitaria, Tacna 23003, Peru; 3 Laboratorio de Biorremediación, Facultad de Ciencias, Universidad Nacional Jorge Basadre Grohmann, Avenida Miraflores S/N, Ciudad Universitaria, Tacna 23003, Peru; 4 Departamento de Biología y Microbiología, Facultad de Ciencias, Universidad Nacional Jorge Basadre Grohmann, Avenida Miraflores S/N, Ciudad Universitaria, Tacna 23003, Peru; 5 Centro de Investigación E-Health, Departamento Ciencias de la Salud, Universidad de Ciencias y Humanidades, Av. Universitaria Norte 5175, Los Olivos Lima 150101, Peru; 6 Laboratorio de Compuestos, Departamento de Ingeniería de Materiales, Facultad de Ingeniería de Procesos, Universidad Nacional de San Agustín de Arequipa, Arequipa 04001, Peru; 7 Laboratorio de Nanotecnología, Facultad de Ingeniería, Universidad Nacional Jorge Basadre Grohmann, Avenida Miraflores S/N, Ciudad Universitaria, Tacna 23003, Peru; 8 Facultad de Ciencias, Universidad Nacional de Ingeniería, Av. Túpac Amaru 210, Lima 15333, Peru

**Keywords:** biogenic synthesis, zinc oxide nanoparticles, glioblastoma, selective cytotoxicity, apoptosis, *Bacillus licheniformis*, green synthesis, cancer therapy

## Abstract

Glioblastoma multiforme (GBM) remains the most aggressive primary brain tumor with median survival of 14.6 months, necessitating novel therapeutic approaches. Here, we report the biogenic synthesis of zinc oxide nanoparticles (ZnO NPs) using *Bacillus licheniformis* strain TT14s isolated from mining environments and demonstrate their selective anti-glioma efficacy. ZnO NPs exhibited hexagonal wurtzite structure (crystallite size: 15.48 nm) with spherical morphology (19.37 ± 5.28 nm diameter) as confirmed by XRD, HRTEM, and comprehensive physicochemical characterization. Colloidal stability analysis revealed an isoelectric point at pH 7.46, ensuring optimal dispersion in biological media. Cytotoxicity evaluation revealed remarkable selectivity: at 100 μg/mL, ZnO NPs reduced NG-108 glioblastoma cell viability to 36.07 ± 1.89% within 1 h while maintaining 78.9 ± 0.94% viability in primary retinal cells. The selective cytotoxicity was attributed to the interplay of convergent mechanisms acting under dark conditions, including defect-mediated ROS generation supported by photoluminescence analysis revealing a characteristic oxygen vacancy emission at 550 nm, pH-dependent dissolution enhanced in the acidic tumor microenvironment, and preferential cellular uptake by rapidly proliferating cancer cells with compromised antioxidant defenses. Time-course analysis demonstrated concentration-dependent effects with therapeutic windows favoring normal cell preservation. The intrinsic cytotoxic activity under dark laboratory conditions eliminates the need for external activation, providing practical advantages for therapeutic applications. These findings establish ZnO NPs as promising candidates for targeted glioblastoma therapy, warranting further in vivo validation and mechanistic elucidation for clinical translation.

## 1. Introduction

Glioblastoma multiforme (GBM) represents the most aggressive and prevalent primary brain malignancy, accounting for approximately 51% of all malignant brain tumors and exhibiting an incidence rate of 3.19–3.26 per 100,000 individuals annually [1,2]. Despite significant advances in neurosurgical techniques, radiotherapy protocols, and chemotherapeutic regimens, GBM remains among the most challenging oncological conditions to treat, with a median overall survival of 14.6 months and a devastating 5-year survival rate of approximately 5–7% [3,4].

The dismal prognosis of GBM stems from several intrinsic biological characteristics that render conventional therapeutic approaches inadequate. Recent breakthrough studies have revealed that glioblastoma cells hijack neuronal mechanisms for brain invasion, exploiting synaptic input and migration pathways that facilitate organized collective invasion through “oncostreams” [5]. This infiltrative behavior, guided by tumor-associated microglia through plexin-B2-mediated alignment, renders complete surgical resection virtually impossible even with maximal safe resection techniques [6]. Furthermore, the heterogeneous tumor microenvironment harbors glioma stem cells (GSCs) that exhibit intrinsic resistance to radiotherapy and chemotherapy, contributing to inevitable tumor recurrence [7,8].

Current standard-of-care treatment, established by the landmark Stupp protocol, combines maximal safe surgical resection followed by concurrent radiotherapy with temozolomide chemotherapy [3]. However, primary resistance to temozolomide affects approximately 50% of patients due to O6-methylguanine-DNA methyltransferase (MGMT) overexpression, while acquired resistance develops through multiple mechanisms including enrichment of treatment-resistant stem cell populations [9,10]. These GSCs, characterized by expression of markers such as CD133, CD44, and SOX2, demonstrate enhanced DNA repair capabilities and altered metabolic profiles that enable survival under therapeutic stress [11].

The blood–brain barrier (BBB) presents an additional formidable obstacle to effective drug delivery, limiting the penetration of therapeutic agents to tumor sites while creating sanctuary sites for residual cancer cells [12,13]. Conventional chemotherapeutic agents often fail to achieve therapeutically relevant concentrations within brain parenchyma, necessitating higher systemic doses that increase toxicity to normal tissues [14].

Active targeting strategies utilizing specific ligand conjugation enhance nanomedicine selectivity beyond passive targeting mechanisms [15]. Peptide-based approaches have demonstrated improved therapeutic indices through receptor-mediated cellular uptake, offering promising strategies for optimizing ZnO nanoparticle selectivity in glioblastoma therapy via brain tumor-specific ligand conjugation.

In recent years, nanotechnology has emerged as a transformative approach in cancer therapeutics, offering unprecedented opportunities to overcome the limitations of conventional treatments [16,17]. Among various nanomaterials, ZnO NPs have garnered significant attention due to their unique physicochemical properties, including biocompatibility, selective cytotoxicity toward cancer cells, and the ability to generate reactive oxygen species (ROS) [18,19]. ZnO NPs exhibit a wide band gap energy of approximately 3.3 eV, enabling photocatalytic activation that enhances their therapeutic efficacy through photodynamic mechanisms [20].

The anticancer activity of ZnO NPs operates through multiple convergent mechanisms, including pH-responsive dissolution in the acidic tumor microenvironment (pH 5.5–6.5), mitochondrial targeting with subsequent apoptosis induction, and selective accumulation in rapidly proliferating cells [18,21]. Studies have demonstrated preferential cytotoxicity against various glioma cell lines (A172, U87, LNZ308) while showing minimal effects on normal astrocytes, suggesting a favorable therapeutic window [22]. The mechanism of selective toxicity involves differential cellular uptake, enhanced ROS generation in cancer cells with compromised antioxidant systems, and zinc ion-mediated disruption of mitochondrial membrane potential [23].

Traditional chemical synthesis methods for ZnO NPs often involve high-temperature processes (500–1000 °C), hazardous chemicals, and significant environmental impact [24]. In contrast, green synthesis approaches utilizing biological organisms offer environmentally sustainable alternatives with enhanced biocompatibility and potentially superior therapeutic properties [25,26]. Bacterial-mediated synthesis, particularly using non-pathogenic species such as *Bacillus licheniformis*, has emerged as a promising approach for producing ZnO NPs with controlled morphology, enhanced stability, and reduced cytotoxicity to normal cells [27,28].

The biogenic synthesis process typically involves extracellular enzyme-mediated reduction of zinc salts through NADH-dependent reductases, with bacterial exopolysaccharides providing natural capping and stabilization [29]. This approach offers several advantages over chemical methods, including ambient temperature and pressure conditions, renewable biological resources, minimal waste generation, and the production of nanoparticles with enhanced colloidal stability and biocompatibility [30,31].

*Bacillus licheniformis*, a Gram-positive, non-pathogenic bacterium widely distributed in natural environments, has demonstrated particular efficacy in nanoparticle biosynthesis due to its robust enzyme systems and ability to secrete stabilizing biomolecules [32]. Previous studies have shown that *B. licheniformis*-mediated synthesis produces ZnO NPs with hexagonal wurtzite crystal structure, optimal size distribution (10–50 nm), and enhanced antimicrobial and anticancer activities compared to chemically synthesized counterparts [33].

The potential of biogenically synthesized ZnO NPs in glioblastoma therapy is particularly noteworthy given their ability to address multiple therapeutic challenges simultaneously. Their nanoscale dimensions may facilitate BBB penetration through transcytosis mechanisms, while their pH-responsive behavior enables selective activation within the acidic tumor microenvironment [34]. Additionally, their capacity to generate multiple ROS species upon UV or visible light activation presents opportunities for photodynamic therapy applications in conjunction with surgical interventions [18].

Recent advances in nanomedicine have demonstrated clinical viability, with over 50 FDA-approved nanotherapeutics currently in clinical use and more than 200 nanomedicine formulations in various stages of clinical trials [35,36]. The translation of ZnO NPs to clinical applications is supported by zinc’s essential biological role (recommended daily intake of 8–11 mg) and its Generally Recognized as Safe (GRAS) status for topical applications [37].

In this study, we aim to evaluate the cytotoxic effects of biogenically synthesized ZnO NPs using *B. licheniformis* on primary cultures of chicken embryonic retinal cells and the NG-108 glioblastoma cell line. By comparing the responses of these two cell types to various concentrations and exposure times of ZnO NPs, we seek to determine the potential for selective cytotoxicity toward glioma cells while preserving normal neural tissue viability.

Our research contributes to the growing body of evidence supporting the development of environmentally sustainable nanotherapeutics for glioblastoma treatment. By elucidating the complex interactions between biogenic ZnO NPs and glioma cells, we aim to establish a foundation for the development of more effective, targeted, and environmentally responsible treatments for this devastating disease, offering new hope for patients suffering from this aggressive form of brain cancer while advancing the principles of green nanotechnology in biomedical applications.

## 2. Materials and Methods

### 2.1. Sample Collection and Bacterial Isolation

The bacterial strain TT14s, employed for ZnO biosynthesis, was originally isolated from soil samples collected at the Tutupaca mining environmental liability site in the Candarave province, Tacna, Peru (coordinates: east 0358602; north 8113940; altitude 4687 m.a.s.l.). The environmental samples underwent systematic homogenization and granulometric separation followed by aqueous extraction under sterile conditions to obtain a cell suspension. The isolation protocol specifically targeted alkaliphilic microorganisms through a selective enrichment strategy, employing sequential subcultures in nutrient broth medium adjusted to pH 10.5 with 0.1 N NaOH. Subsequently, discrete bacterial colonies exhibiting alkaline-tolerance were isolated through streak-plate technique on nutrient agar under identical pH conditions. The isolate was preserved in the Bioremediation Laboratory culture collection at Jorge Basadre Grohmann National University, where it was assigned the designation TT14s for taxonomic and experimental purposes.

### 2.2. Molecular Identification and Sequencing

DNA extraction from bacterial isolate TT14s was accomplished employing the InnuPREP Bacteria DNA Kit according to the protocol provided by the manufacturer. Quantitative and qualitative assessment of the extracted DNA was performed through fluorescence-based measurement using a Qubit 4 fluorometer (Life Technologies, Carlsbad, CA, USA).

Sequencing library construction was carried out using the Illumina DNA Prep protocol (Illumina, Granta Park, Cambridge, UK), with incorporation of distinctive dual barcodes from the Nextera DNA CD Indexes system (Illumina, Granta Park, Cambridge, UK) to facilitate sample multiplexing during sequencing runs. Whole-genome sequencing was performed on the Illumina MiSeq Control platform (Version 2.6.0) employing a V3 600-cycle chemistry kit, which produced paired-end sequence reads of 2 × 151 nucleotides.

Species-level identification was achieved through phylogenetic analysis of the 16S ribosomal RNA gene sequence recovered from the complete genome assembly. Taxonomic assignment was determined by sequence homology searching against the National Center for Biotechnology Information (NCBI) reference database using the Basic Local Alignment Search Tool (BLAST+, Version 2.15.0) algorithm. This genomic approach for bacterial identification, consistent with methodologies established by Cáceda et al. (2023) [38], enabled precise taxonomic placement of isolate TT14s.

### 2.3. Biogenic ZnO Synthesis by Bacillus licheniformis

The green synthesis of ZnO nanoparticles was conducted using *Bacillus licheniformis* strain TT14s, previously isolated and molecularly characterized from our laboratory’s microbial repository. The synthetic approach was adapted from established protocols reported by Tripathi and colleagues (2021) [39], incorporating specific modifications to optimize production yield and scalability. The experimental procedure initiated with bacterial reactivation in Brain Heart Infusion (BHI) medium at 35 °C for 24 h. Following reactivation, cultures were transferred onto nutrient agar plates and incubated at 35 °C for an additional 24 h period to obtain viable bacterial colonies. Morphological verification was conducted through Gram staining techniques to confirm the characteristic features of *B. licheniformis*: Gram-positive, rod-shaped bacterial cells with terminal rounding and endospore-forming capability.

Active bacterial cultures were grown in nutrient broth at 35 °C with continuous agitation at 180 rpm over a 48 h period. Biomass collection was achieved through centrifugation at 8000 rpm for 10 min. The biosynthetic reaction was initiated by combining 5 g of harvested bacterial biomass with a precursor solution containing 375 mL of 0.2 M zinc acetate dihydrate and 375 mL of 0.5 M sodium bicarbonate, both dissolved in sterile deionized water. The reaction vessel was maintained at 35 °C under continuous agitation at 170 rpm for 72 h to promote bacterial-mediated nanoparticle formation.

Product recovery involved centrifugal separation at 8000 rpm for 10 min, followed by six consecutive washing steps using sterile deionized water to eliminate impurities and residual bacterial byproducts. The recovered precipitate underwent thermal dehydration at 60 °C for 18 h. Thermal analysis and crystallization enhancement were conducted using thermogravimetric and differential scanning calorimetric techniques. Final crystallization was accomplished through mechanical homogenization using an agate pestle and mortar, followed by calcination at 350 °C for 2 h in a ceramic vessel. A final grinding step was performed to deaggregate the calcined ZnO nanoparticles, producing a uniform powder suitable for comprehensive characterization and biological evaluation.

### 2.4. Characterization Techniques

Thermal characterization of the biosynthesized ZnO nanoparticles was conducted using multiple complementary analytical approaches. Weight loss behavior and thermal transitions were evaluated through thermogravimetric analysis (TGA) coupled with differential scanning calorimetry (DSC) on samples prior to calcination, employing an SDT650 thermal analyzer (TA Instruments, New Castle, DE, USA) operating under nitrogen atmosphere (100 mL/min flow rate) with a controlled heating ramp of 20 °C/min. Crystallographic structure determination was accomplished via X-ray powder diffraction (XRD) analysis of calcined specimens using a PANalytical Aeris Research diffractometer operating with Cu Kα radiation at 40 kV and 15 mA (Malvern Panalytical Ltd., Almelo, The Netherlands). Vibrational spectroscopic characterization was performed using attenuated total reflectance Fourier-transform infrared (ATR-FTIR) analysis across the 200–4000 cm^−1^ spectral range with a Bruker Invenio R spectrometer (Ettlingen, Germany). Morphological assessment was conducted through transmission electron microscopy (TEM) employing a Thermo Scientific Talos 200i instrument operating at 200 kV (Thermo Scientific Co., Eindhoven, The Netherlands). Surface topography investigation was carried out using scanning electron microscopy with a Thermo Scientific Quattro S microscope at 30 kV under high vacuum conditions (Thermo Scientific Co., Eindhoven, The Netherlands). Photoluminescence (PL) measurements were performed using a fluorescence spectrometer (Fluorormax Plus, Horiba Scientific, Irvine, CA, USA) at room temperature with multiple excitation wavelengths (325, 365, and 405 nm) to comprehensively analyze defect-related emission characteristics. The defect-related emission intensity was correlated with potential ROS generation capacity based on established literature relationships between oxygen vacancy defects and reactive species formation.

### 2.5. Zeta Potential and Colloidal Stability Assessment

Zeta potential measurements were conducted using the salt addition method to determine the isoelectric point and assess colloidal stability. Solutions containing 50 mg ZnO nanoparticles in 50 mL of 0.1 M NaCl were prepared at pH values ranging from 3 to 11, adjusted using 0.1 M HCl and NaOH solutions. Samples were maintained under constant stirring for 24 h at 25 °C and subsequently filtered through 0.45 μm membrane filters, and the final pH was measured using a calibrated pH meter. The isoelectric point was determined from the intersection of initial vs. final pH plots. Chemical stability was verified by X-ray diffraction analysis of samples recovered after pH stability studies.

### 2.6. Primary Cultures of Embryonic Chicken Retinal Neuronal Cells

Fertilized chicken eggs (*Gallus gallus*, White Leghorn breed) at embryonic development stage 7 (E7) were sourced from a local commercial supplier (Arequipa, Peru) and maintained in controlled incubation at 37.4 °C with 5% moisture content. Embryonic tissue extraction was conducted under aseptic conditions within a laminar airflow workstation. After rapid enucleation of ocular structures, eyes were transferred to 40 mm glass culture vessels containing calcium and magnesium-free saline buffer (CMF). Retinal tissues were carefully extracted and subjected to enzymatic disaggregation using 0.2% trypsin solution (Sigma-Aldrich, St. Louis, MO, USA) for 15 min at 37 °C. Post-dissociation, cellular material was cultured in Dulbecco’s Modified Eagle Medium (DMEM, Invitrogen, Carlsbad, CA, USA) supplemented with 10% fetal bovine serum (FBS, Sigma-Aldrich, St. Louis, MO, USA) and antimicrobial agents comprising 100 U/mL penicillin and 100 μg/mL streptomycin (Invitrogen, USA). Cellular suspensions were distributed onto 35 mm culture vessels (Corning, Corning, NY, USA) at a plating density of 2.5 × 10^6^ cells per dish. Incubation conditions were maintained at 37 °C in a humidified environment containing 5% CO_2_ and 95% atmospheric air.

### 2.7. NG-108 Glioblastoma Cell Line Culture

The NG-108 cellular model, representing a fusion hybrid of mouse N18TG-2 neuroblastoma and rat C6 BV-1 glioma lineages, was utilized in this investigation. Cryopreserved NG-108 stocks (comprising 40% DMEM, 50% FBS, 10% DMSO) were rapidly thawed at 37 °C in a water bath. Cellular material was gently resuspended and transferred to 10 mL culture tubes containing 1 mL DMEM medium. Cell lines were established in DMEM medium supplemented with 2 mM L-glutamine (Sigma-Aldrich, USA) and 10% FBS. Following 24 h of initial culture, the growth medium was refreshed to eliminate residual DMSO. Cellular maintenance was performed in 25 cm^2^ culture flasks (Corning, USA) at 37 °C with 5% CO_2_ atmosphere for extended periods. Medium renewal was conducted every 48 h. Subculturing procedures were executed at 70% confluency using 0.25% trypsin-EDTA solution (Gibco, USA). Cell suspensions underwent centrifugation at 3500 rpm for 5 min, and cellular quantification was accomplished using a Neubauer hemocytometer.

### 2.8. Zinc Oxide NP Concentrations and Treatment Times

Primary retinal cell cultures (E7C2) were maintained in DMEM medium supplemented with 10% FBS, 100 U/mL penicillin, and 100 μg/mL streptomycin at 37 °C in a 5% CO_2_ humidified environment. ZnO nanoparticles underwent sterilization through autoclave treatment at 121 °C for 10 min at 15 psi pressure. A master stock solution of 1 mg/mL ZnO nanoparticles was prepared in sterile ultrapure water. Before cellular treatment, nanoparticles were subjected to ultrasonic dispersion (Branson Ultrasonics, Danbury, CT, USA) at ambient temperature for 10 min to ensure uniform distribution. Embryonic retinal and NG-108 cell cultures were exposed to ZnO nanoparticles at final working concentrations of 10, 50, and 100 μg/mL for exposure periods of 1, 6, 12, and 24 h. Control cultures were maintained without nanoparticle exposure for each experimental condition.

### 2.9. MTT Cell Viability Assays

Cytotoxic effects of ZnO nanoparticles were evaluated using the MTT (3-(4,5-Dimethylthiazol-2-yl)-2,5-diphenyltetrazolium bromide) colorimetric viability assay. Embryonic retinal cellular preparations (2.5 × 10^6^ cells/dish) and NG-108 glioblastoma cultures (1 × 10^6^ cells/20 mm dish) underwent treatment with ZnO nanoparticles as previously described. Post-treatment, MTT reagent (5 mg/mL in PBS, Sigma-Aldrich, USA) was introduced to each culture well and maintained for 4 h at 37 °C. Subsequently, cultures underwent washing with Hank’s Balanced Salt Solution (HBSS composition: NaCl 128 mM, KCl 4 mM, Na_2_HPO_4_ 1 mM, KH_2_PO_4_ 0.5 mM, MgCl_2_ 1 mM, CaCl_2_ 3 mM, HEPES 20 mM, glucose 12 mM, pH 7.4). Formazan crystal dissolution was accomplished using an isopropanol-HCl mixture (1:0.6 ratio). Optical density measurements were performed at 570 nm using a spectrophotometer (Ultrospec 1000, Pharmacia Biotech, Amersham, UK).

### 2.10. DAPI Nuclear Staining in NG-108 Glioma Cultures

NG-108 cellular cultures exposed to ZnO nanoparticles (10, 50, and 100 μg/mL) for 1 h underwent nuclear morphology assessment. Culture preparations were washed twice with phosphate-buffered saline (0.16 M, pH 7.6) and fixed using 4% paraformaldehyde in PBS for 15 min at ambient temperature. Nuclear visualization was performed using DAPI (4′,6-diamidino-2-phenylindole, Invitrogen, USA) following the manufacturer’s protocols. Cellular specimens were observed and documented using an Olympus IX73 Inverted Fluorescence Microscope (Olympus, Tokyo, Japan). Quantification of cells displaying normal versus fragmented nuclear morphology was conducted across 6 randomly selected fields at 37.8× magnification.

### 2.11. Statistical Analysis

Data analysis was performed using GraphPad Prism 8.4 statistical software (GraphPad Software Inc., San Diego, CA, USA). All experimental results are reported as mean values ± standard error of the mean (SEM) calculated from a minimum of three independent experimental replicates. Statistical significance evaluation was conducted through one-way analysis of variance (ANOVA), with post hoc analysis utilizing Tukey’s honestly significant difference test and Dunnett’s multiple comparison methodology. Statistical significance threshold was established at *p* < 0.05.

## 3. Results

### 3.1. Results of Molecular Identification

The taxonomic identification of bacterial strain TT14s was conducted through comprehensive molecular analysis of the 16S rRNA gene sequence using high-resolution comparative genomics approaches. The amplified sequence, comprising 1242 base pairs, was subjected to nucleotide BLAST analysis against the curated NCBI GenBank database to establish phylogenetic relationships. Sequence similarity analysis revealed 100% sequence identity with reference strains of *Bacillus licheniformis* (GenBank accession numbers NR_118996.1, NR_116023.1), indicating precise taxonomic placement within this species. To establish evolutionary relationships and confirm the taxonomic position, phylogenetic reconstruction was performed using the Neighbor Joining algorithm, incorporating appropriate nucleotide substitution models and statistical parameters. The resulting phylogenetic tree topology unambiguously positioned strain TT14s within the *B. licheniformis* clade, providing robust support for its taxonomic classification. This molecular systematic approach, based on 16S rRNA gene sequence analysis, represents a reliable method for bacterial identification, particularly within the genus Bacillus, where this genetic marker exhibits sufficient discriminatory power for species-level resolution. For reference purposes and to facilitate future comparative analyses, the 16S rRNA gene sequence of strain TT14s has been deposited in the GenBank nucleotide database and assigned the accession number PV034281.

### 3.2. Thermogravimetric and Scanning Differential Calorimetry

**Figure 1:**
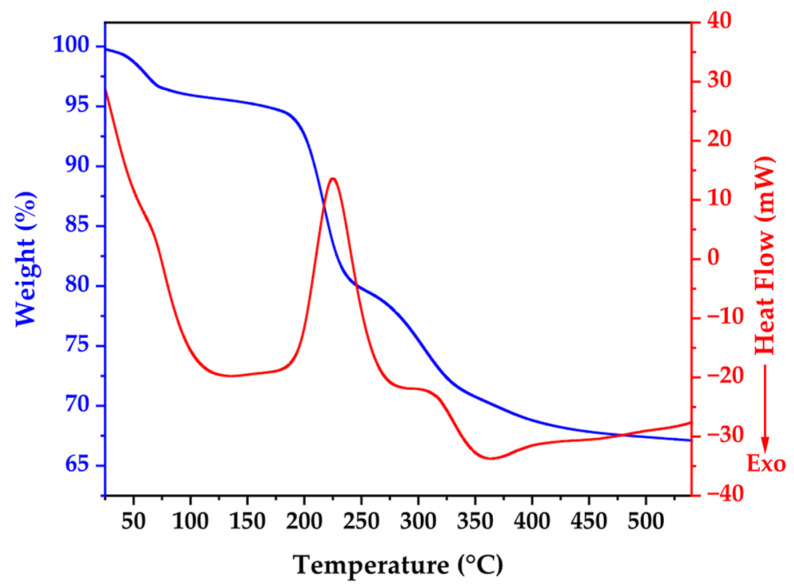
Thermogravimetric and differential scanning calorimetry of as prepared sample.

Figure 1 presents the thermal behavior of biosynthesized ZnO NPs through combined TGA-DSC analysis, revealing critical insights into material composition and transformation processes. The TGA curve demonstrates gradual mass reduction from ambient temperature, with pronounced weight loss between 200 °C and 300 °C, accounting for approximately 33% of initial sample weight, confirming substantial organic content from bacterial synthesis.

The DSC profile exhibits five distinct thermal events with specific mechanistic significance. An initial endothermic peak at 139.58 °C is attributed to desorption of surface-bound water molecules and volatile organic compounds from bacterial cell walls. A subsequent endothermic transition at 220 °C corresponds to zinc acetate complex decomposition and bacterial protein degradation. The major endothermic event at 285 °C represents primary bacterial biomass and organic capping agent decomposition, coinciding with maximum TGA mass loss. An exothermic peak at 365 °C indicates ZnO crystallization and annealing processes, while thermal events beyond 419 °C signify final organic matter elimination and crystal structure optimization.

The thermal profile stabilization beyond 350 °C validates the chosen calcination parameters (350 °C for 2 h), confirming effective removal of bacterial remnants while facilitating high-quality crystalline ZnO formation. This systematic thermal analysis elucidates the crucial role of controlled thermal treatment in transforming biogenic precursors into pure, crystalline ZnO nanoparticles with optimized structural properties [40,41].

### 3.3. X-Ray Diffraction Results

**Figure 2:**
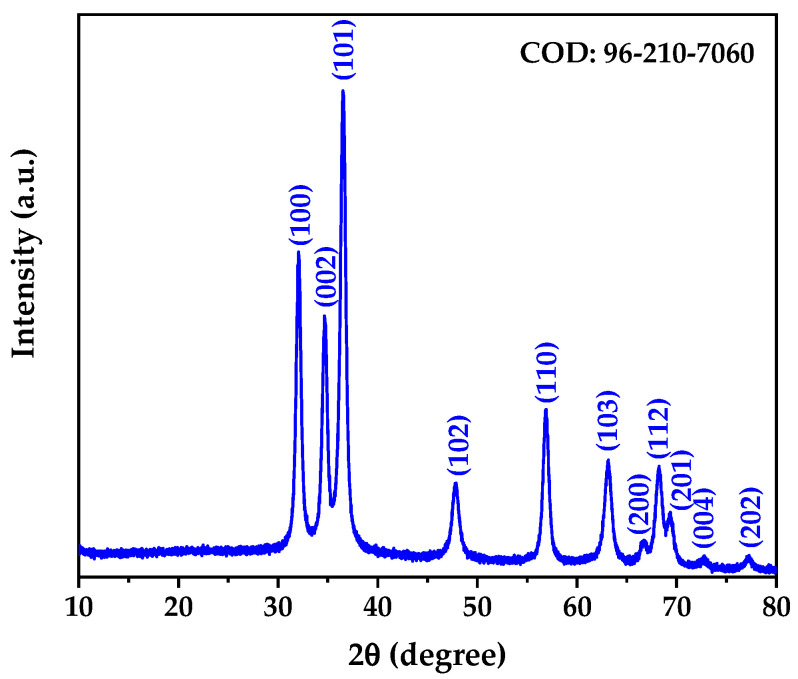
X-ray diffraction of obtained material.

The X-ray diffraction pattern of the biogenically synthesized ZnO NPs is presented in Figure 2. The diffractogram reveals well-defined diffraction peaks that can be indexed to the hexagonal wurtzite crystal structure of zinc oxide, consistent with the Crystallography Open Database (COD) reference 96-210-7060. All observed diffraction peaks at 2θ values of 32.03°, 34.67°, 36.51°, 47.78°, 56.83°, 63.07°, 68.16°, 69.30°, 72.76°, and 77.16° correspond to the (100), (002), (101), (102), (110), (103), (112), (201), (004), and (202) crystallographic planes, respectively, confirming the formation of phase-pure ZnO without detectable impurities [42].

Rietveld structure refinement was performed to obtain precise crystallographic parameters and structural information. The refinement confirmed that the synthesized ZnO NPs crystallize in the hexagonal crystal system with space group P 63 m c (No. 186), characteristic of the wurtzite structure [43]. The refined lattice parameters were determined to be a = b = 3.25018 Å and c = 5.2087 Å, yielding a c/a ratio of 1.603, which is consistent with the ideal wurtzite structure. These values are in excellent agreement with the COD Card 96-210-7060 standard reference for synthetic wurtzite.

The quality of the Rietveld refinement was assessed through statistical reliability factors. The weighted profile R-factor (R_wp_) of 4.61% and profile R-factor (R_p_) of 3.36% indicate an excellent fit between the experimental and calculated diffraction patterns [44]. The goodness of fit (GOF) value of 0.94 confirms the reliability of the structural model, as values close to unity indicate optimal refinement quality [45].

The average crystallite size was determined directly from the Rietveld refinement through the analysis of peak broadening parameters, yielding a value of 15.48 nm. This microstructural parameter was extracted from the refined profile function that accounts for both instrumental and sample-dependent broadening effects, providing a more accurate determination compared to single-peak analysis methods [46]. The obtained crystallite size falls within the typical range reported for biogenically synthesized ZnO NPs, confirming the nanocrystalline nature of the synthesized material [47].

Microstrain analysis revealed a low microstrain value of 0.016%, indicating minimal lattice distortions in the crystal structure. This low microstrain suggests well-ordered crystalline domains with minimal defects, which is characteristic of high-quality ZnO NPs synthesized under controlled conditions [48]. The calculated theoretical density of 5.67 g/cm^3^ matches the standard bulk density of ZnO, confirming the formation of dense crystalline nanoparticles without significant porosity or structural defects.

The sharp and well-resolved diffraction peaks, combined with the absence of additional peaks, confirm the formation of single-phase hexagonal ZnO with high crystallinity. The narrow peak widths indicate good structural ordering and uniform crystallite size distribution, which is advantageous for potential biomedical applications where consistent physicochemical properties are crucial [49].

### 3.4. FTIR Spectroscopy Results

**Figure 3:**
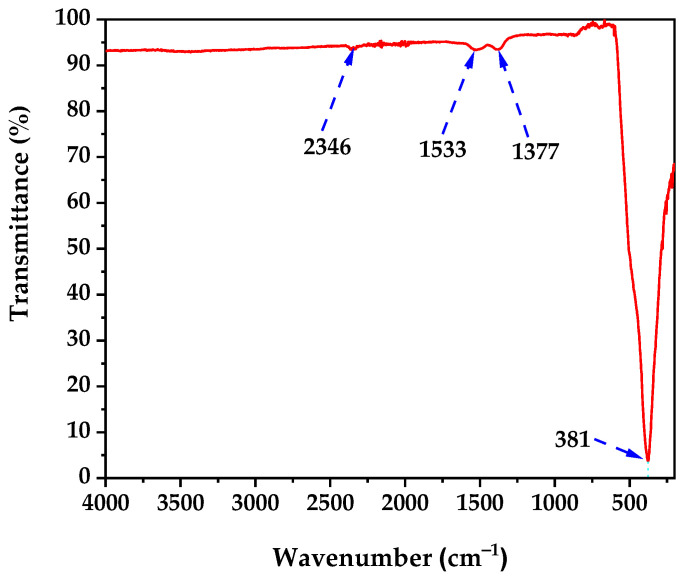
FTIR spectra of ZnO nanoparticles.

The FTIR spectrum of the calcined ZnO NPs (Figure 3) confirms successful biogenic synthesis and thermal processing. The spectrum exhibits four distinct absorption bands at 2346, 1533, 1377, and 381 cm^−1^, which validate both ZnO formation and controlled surface chemistry following the 350 °C calcination treatment.

The broad transmission baseline above 3000 cm^−1^ indicates minimal O-H stretching vibrations from surface hydroxyl groups, confirming effective removal of bacterial biomass and adsorbed water during calcination [50]. This reduced hydration is consistent with the 33% mass loss observed in TGA analysis and demonstrates successful thermal processing.

The sharp absorption band at 2346 cm^−1^ corresponds to atmospheric CO_2_ contamination during spectroscopic measurement [51]. However, minor contributions may arise from residual carbonate species formed during the synthesis process involving sodium bicarbonate as a co-precursor.

The absorption band at 1533 cm^−1^ is assigned to N-H bending vibrations and C-N stretching modes from thermally stable nitrogen-containing compounds, likely originating from bacterial cell wall components that survived the calcination process, indicating selective preservation of specific biomolecular functionalities that may contribute to enhanced biocompatibility. Additionally, the band at 1377 cm^−1^ corresponds to symmetric stretching vibrations of carboxylate groups (COO^−^), formed during the interaction between zinc acetate precursors and bacterial exudates during the 72-h incubation period [52,53].

The most diagnostic absorption band appears at 381 cm^−1^, corresponding to the characteristic Zn-O stretching vibrations of the hexagonal wurtzite crystal structure [54]. The sharp, intense nature of this fundamental vibration confirms formation of highly crystalline ZnO NPs, validating the effectiveness of the thermal treatment in promoting complete crystallization while maintaining the desired wurtzite structure.

The FTIR spectrum demonstrates that the biogenic synthesis protocol using *B. licheniformis* followed by 350 °C calcination produces ZnO NPs with minimal organic surface modification. The thermal treatment effectively removed the majority of bacterial biomass while preserving only essential surface functionalities (carboxylate and amine groups) that enhance biocompatibility without compromising the intrinsic crystalline properties of ZnO [25]. This controlled surface chemistry represents an optimal balance for biomedical applications, maintaining the crystalline integrity necessary for anticancer activity while preserving selective organic modifications derived from the bacterial synthesis process.

### 3.5. Scanning and Transmission Electron Microscopy

**Figure 4:**
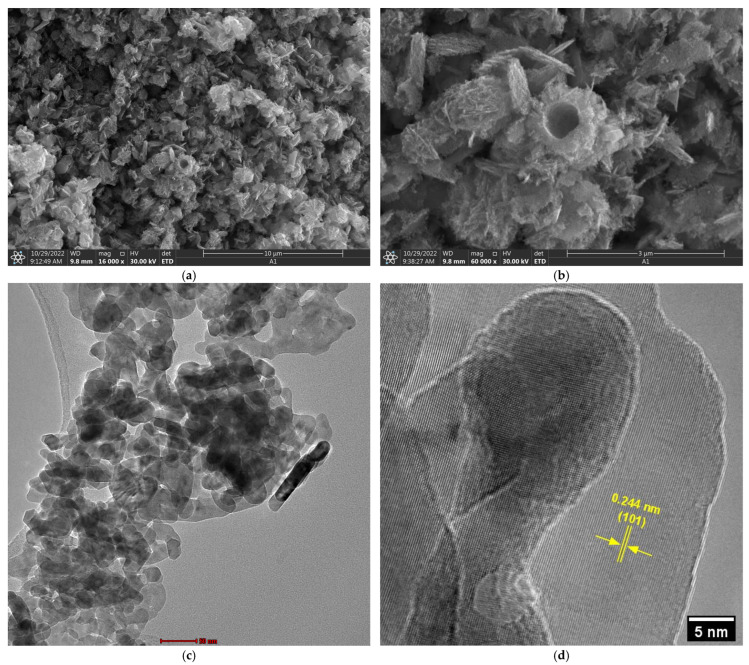
Electron microscopy characterization of biogenic ZnO NPs. (**a**,**b**) Scanning Electron Microscopy images at different magnifications showing spherical and plate-like ZnO structures. (**c**) TEM image revealing individual nanoparticles with average size of 19.37 ± 5.28 nm forming the observed structures. (**d**) HRTEM image showing lattice fringes with d-spacing of 0.244 nm corresponding to the (101) plane of wurtzite ZnO.

Scanning electron microscopy analysis (Figure 4a,b) revealed that biogenic ZnO NPs exhibit a complex morphological architecture characterized by primary structures ranging from spherical to plate-like configurations. These secondary structures comprise aggregates of individual nanoparticles, as clearly evidenced in transmission electron microscopy (TEM) images (Figure 4c), where apparently compact structures are observed to consist of discrete nanoparticles with an average size of 19.37 ± 5.28 nm. This hierarchical organization is characteristic of biogenic synthesis, wherein biomolecules function as stabilizing and morphology-directing agents, promoting the formation of controlled aggregates rather than isolated particles [55,56].

High-resolution transmission electron microscopy (HRTEM) (Figure 4d) analysis confirmed the crystalline nature of the synthesized material, revealing an interplanar spacing of 0.244 nm corresponding to the (101) crystallographic plane of the wurtzite ZnO structure [57]. This measurement demonstrates excellent agreement with reported values for high-quality crystalline ZnO and confirms that the biogenic synthesis process does not compromise the structural integrity of the material, maintaining the crystallographic properties necessary for advanced applications [58].

### 3.6. Photoluminescence Analysis and Defect Characterization

**Figure 5:**
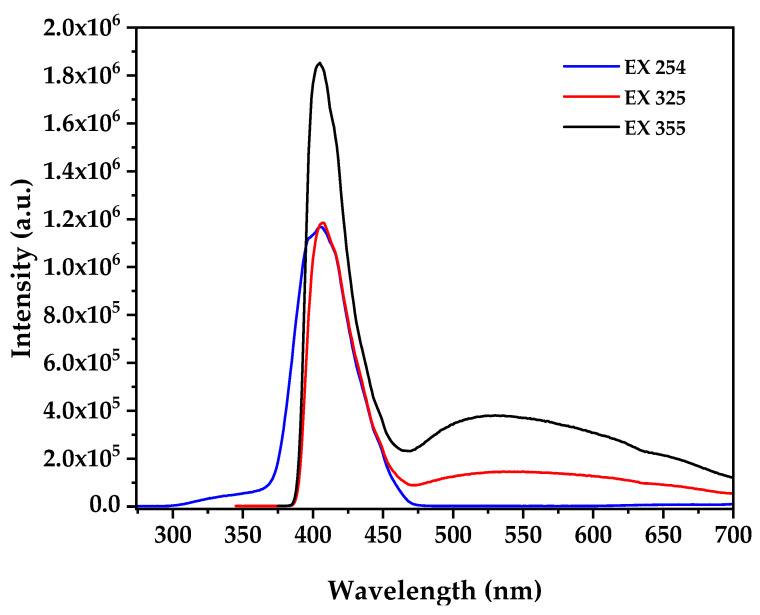
PL Spectra of biogenic ZnO NPs at distinct excitation wavelengths.

Photoluminescence spectroscopy at multiple excitation wavelengths provides critical insights into the electronic defect structure correlating with biological activity (Figure 5), where the PL spectra at excitation wavelengths of 254, 325, and 355 nm reveal distinct emission characteristics that depend on the excitation energy and penetration depth, with 254 nm excitation (4.88 eV) showing a dominant sharp emission peak centered at approximately 380 nm attributed to near-band-edge (NBE) recombination of free excitons consistent with established ZnO nanostructure while 325 nm excitation [59] (3.82 eV) produces NBE emission at 380 nm accompanied by broad defect-related emission extending from 500–600 nm with moderate intensity, and most significantly, 355 nm excitation (3.49 eV) generates the most intense broad emission band centered at approximately 550 nm in the green-yellow region accompanied by a secondary defect band around 500 nm. The broad emission at 550 nm observed under 355 nm excitation is attributed to oxygen vacancy (V_o_) and zinc interstitial (Zn_i_) defects within the ZnO lattice formed during the biogenic synthesis process under reducing bacterial metabolite conditions, where the excitation wavelength dependence indicates different defect levels being accessed, with 254 nm primarily activating band-edge states with minimal defect activation, 325 nm accessing intermediate defect levels with moderate oxygen vacancy contribution, and 355 nm achieving maximum defect state population particularly oxygen vacancies [60]. According to established literature correlations, the intensity of defect emission under 355 nm excitation directly correlates with ROS generation capacity under dark conditions through defect-mediated mechanisms where oxygen vacancies interact with water molecules to form hydroxyl radicals and subsequently generate superoxide anions and hydrogen peroxide in aqueous cellular environments through the reaction sequence [61] $V_{O}+H_{2}O\to V_{O}^{+}+{OH}^{-}+e^{-}$, followed by $e^{-}+O_{2}\to O_{2}^{-•}$ and ${2O}_{2}^{-•}+2H^{+}\to H_{2}O_{2}+O_{2}$. The high defect-to-band-edge emission ratio indicates substantial oxygen vacancy concentration explaining the observed cytotoxic activity through defect-mediated ROS generation in aqueous cellular environments without external photoactivation, where this intrinsic ROS generation capacity maximally activated under near-UV conditions (355 nm) provides the mechanistic foundation for the selective anticancer activity observed in biological assays, as demonstrated by studies showing direct correlation between photoluminescence defect emission intensity and enhanced reactive oxygen species production capacity in ZnO nanoparticles with formation energies of oxygen vacancies calculated as 1.3–1.52 eV using thermodynamic approaches [21,62].

### 3.7. Colloidal Stability and Surface Charge Characteristics

**Figure 6:**
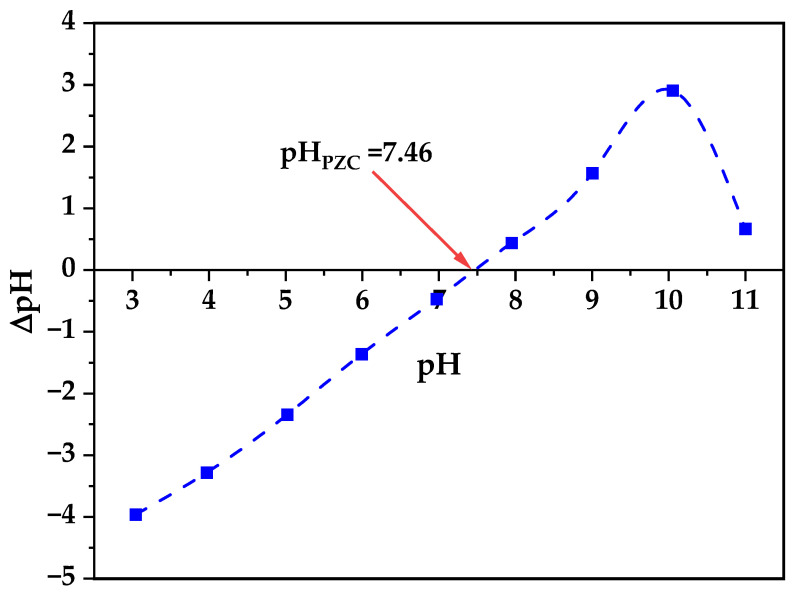
Zeta Potential of biogenic ZnO.

Zeta potential analysis reveals critical information about colloidal stability and surface interactions relevant to biological applications (Figure 6), where the zeta potential varies systematically with pH exhibiting an isoelectric point (IEP) at pH 7.46 that is particularly significant as it closely matches physiological pH conditions (7.4), resulting in near-neutral surface charge (±2.5 mV) during biological assays, which differs from typical literature values of pH 9.7–10.3 for pristine ZnO nanoparticles indicating substantial surface modification effects from the biogenic synthesis process involving bacterial metabolites and extracellular polymeric substances [63]. At the experimental pH conditions used for cytotoxicity studies (pH 7.4–7.6), the minimal surface charge indicates reduced electrostatic interactions with cell membranes enabling controlled cellular uptake, minimal protein corona formation preserving nanoparticle bioactivity, and optimal dispersion stability without excessive aggregation or repulsion, as established by studies demonstrating that surface neutrality achieved through natural capping agents significantly limits binding sites for undesired serum proteins thereby reducing immune system activation and improving bioavailability in biological systems [64].

**Figure 7:**
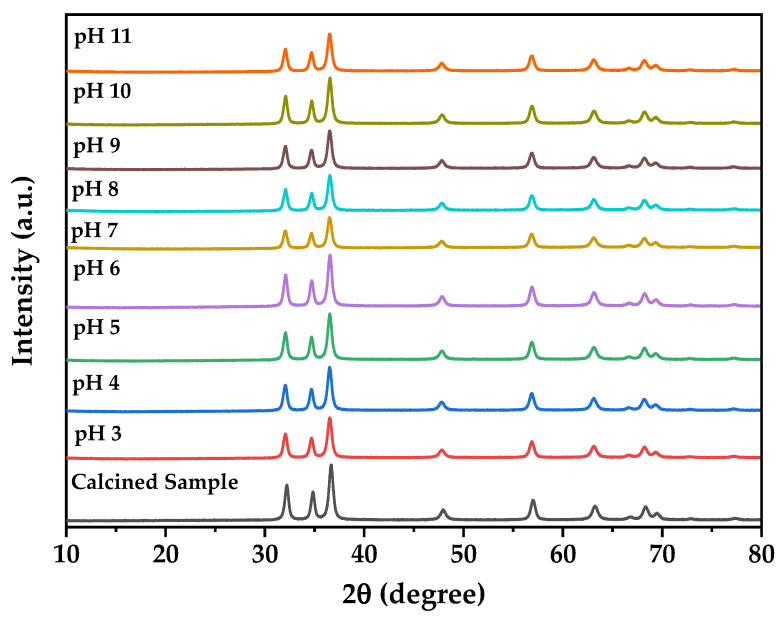
XRD analysis after different pH exposure of biogenic ZnO.

The pH stability study demonstrates remarkable chemical robustness across the tested pH range (3–11) where XRD analysis of samples recovered after 24 h exposure to extreme pH conditions (Figure 7) confirms retention of the pure hexagonal wurtzite structure without phase transitions or decomposition products, validating the suitability of biogenic ZnO nanoparticles for diverse biological applications spanning different physiological pH environments, which is consistent with reports showing ZnO nanoparticles maintain structural integrity despite size-dependent dissolution at circumneutral pH where organic chelators can enhance dissolution for smaller particles while larger aggregates remain stable [65]. This stability profile combined with the near-neutral zeta potential at physiological pH optimizes the balance between cellular uptake efficiency and biocompatibility contributing to the observed selective cytotoxicity profile against glioblastoma cells, where the enhanced stability compared to chemically synthesized ZnO is attributed to the protective biomolecular capping layer formed during bacterial synthesis providing steric stabilization and preventing aggregation-induced precipitation [66,67,68].

### 3.8. Effect of ZnO NPs on Primary Retinal Cells

**Figure 8:**
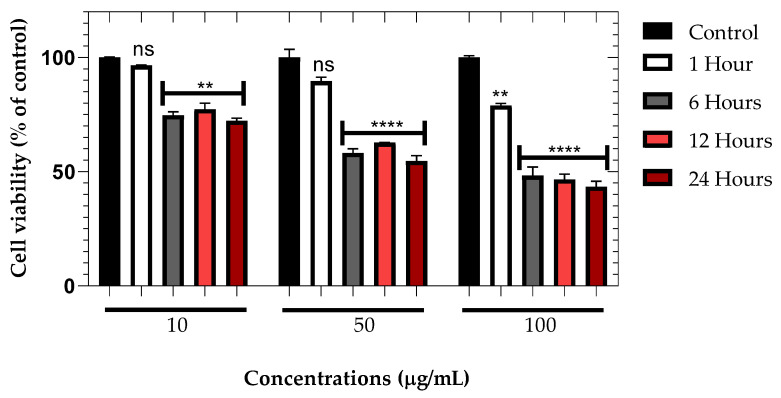
Time- and concentration-dependent cytotoxicity of ZnO NPs on primary retinal cells. Data are presented as mean ± SEM. ** *p* < 0.01, **** *p* < 0.0001 compared to control (one-way ANOVA followed by Dunnett’s multiple comparisons test).

The cytotoxic effect of ZnO NPs on primary cultures of embryonic chicken retinal cells was evaluated using the MTT assay. Our results demonstrated a concentration- and time-dependent cytotoxicity (Table 1, Figure 8). At lower concentrations (10 and 50 μg/mL), no significant cytotoxicity was observed after 1 h of incubation (96.53 ± 0.22% and 89.68 ± 1.70% viability, respectively; *p* > 0.05). However, significant cell death was observed at these concentrations after 6, 12, and 24 h of exposure (*p* < 0.001).

At the highest concentration tested (100 μg/mL), significant cytotoxicity was observed at all time points. Cell viability decreased to 78.90 ± 0.94% after 1 h and further declined to 43.48 ± 2.34% after 24 h of exposure (*p* < 0.0001). These results indicate that primary retinal cells are relatively resistant to short-term exposure to ZnO NPs at lower concentrations but become increasingly susceptible over time and at higher concentrations.

The observed time-dependent increase in cytotoxicity suggests a cumulative effect of ZnO NPs on retinal cells. This could be due to gradual nanoparticle uptake, sustained generation of reactive oxygen species (ROS), or progressive disruption of cellular processes. The concentration-dependent effect indicates a dose-threshold for significant cytotoxicity, which could be important for determining safe exposure levels in potential therapeutic applications [69,70,71].

### 3.9. Effect of ZnO NPs on NG-108 Glioblastoma Cells

**Figure 9:**
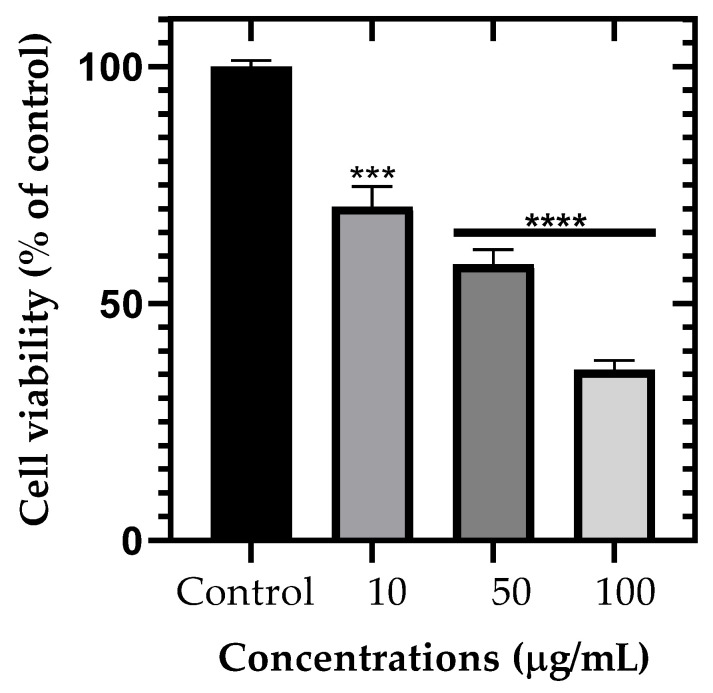
Dose-dependent cytotoxicity of ZnO NPs on NG-108 glioblastoma cells after 1 h of exposure. Data are presented as mean ± SEM. *** *p* < 0.001, **** *p* < 0.0001 compared to control (one-way ANOVA followed by Dunnett’s multiple comparisons test).

The cytotoxic effect of ZnO NPs on NG-108 glioblastoma cells was more pronounced compared to primary retinal cells (Table 2, Figure 9). After just 1 h of incubation, significant reductions in cell viability were observed across all tested concentrations. Cell viability decreased to 70.41 ± 4.29%, 58.23 ± 3.15%, and 36.07 ± 1.89% at 10, 50, and 100 μg/mL, respectively (*p* < 0.001 for 10 μg/mL and *p* < 0.0001 for 50 and 100 μg/mL).

These results demonstrate a higher sensitivity of NG-108 glioblastoma cells to ZnO NPs compared to primary retinal cells, particularly at shorter exposure times. This differential cytotoxicity suggests a potential therapeutic window for targeting glioblastoma cells while minimizing damage to healthy neural tissue. The rapid onset of cytotoxicity in glioblastoma cells could be attributed to their higher metabolic rate, increased proliferation, or altered membrane permeability compared to normal cells [72,73,74].

### 3.10. Comparative Analysis of ZnO NPs’ Sensitivity

**Figure 10:**
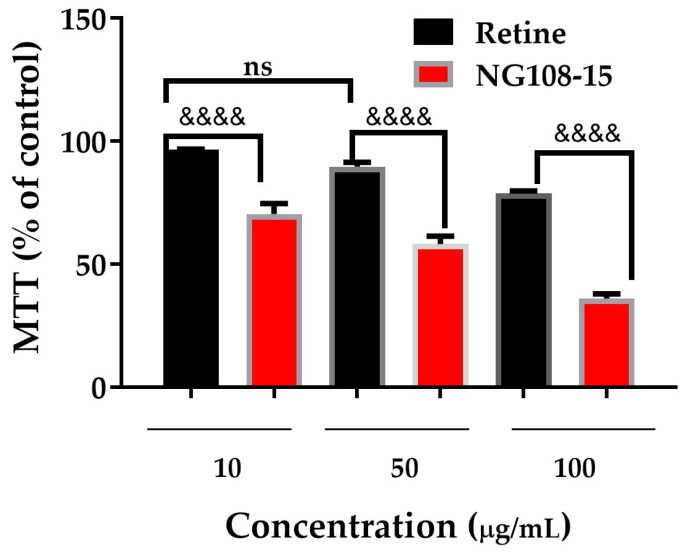
Comparative cell viability of retinal cells and NG-108 glioblastoma cells exposed to ZnO NPs for 1 h. Data represent mean ± SEM (n = 3–4). Two-way ANOVA with Sidak’s post hoc test. &&&& *p* < 0.0001 between cell types; ns = not significant.

To quantitatively assess the differential cytotoxic response and establish the therapeutic window of ZnO NPs, we performed a comprehensive comparative analysis be-tween primary retinal cells and NG-108 glioblastoma cells after 1 h of exposure (Figure 10). Two-way ANOVA revealed highly significant main effects of both cell type (F_1,14_ = 89.42, *p* < 0.0001) and ZnO concentration (F_3,14_ = 11.00, *p* < 0.001), with no significant interaction effect (F_3,14_ = 1.25, *p* = 0.326), indicating that the differential sensitivity pattern remains consistent across all tested concentrations.

Post hoc analysis using Sidak’s multiple comparisons test revealed a concentration-dependent pattern of selectivity: no significant difference between cell types at 10 μg/mL (ns), but highly significant differences at 50 μg/mL (*p* < 0.0001, mean difference: 40%) and 100 μg/mL (*p* < 0.0001, mean difference: 45%). These results demonstrate that selective cytotoxicity emerges at concentrations ≥ 50 μg/mL, establishing a clear threshold for therapeutic selectivity.

The concentration-dependent selectivity analysis revealed minimal selectivity at 10 μg/mL (1.4-fold, ns), but substantial therapeutic windows at higher concentrations: 1.7-fold at 50 μg/mL and 2.3-fold at 100 μg/mL (both *p* < 0.0001). Notably, NG-108 glioblastoma cells exhibited significant cytotoxicity at all concentrations compared to un-treated controls (*p* < 0.001 for all concentrations), whereas primary retinal cells showed significant viability reduction only at 100 μg/mL (*p* < 0.05), confirming the safety profile for normal neural tissue at therapeutically relevant concentrations.

The rapid onset of selective cytotoxicity within 1 h suggests efficient cellular up-take and immediate biological activity in cancer cells, likely mediated by enhanced membrane permeability and compromised antioxidant systems characteristic of malignant phenotypes. This temporal pattern, combined with the dose-dependent selectivity profile, positions ZnO NPs as promising candidates for targeted glioblastoma therapy with minimal off-target effects on healthy neural tissue [19,24,69].

### 3.11. Nuclear Morphology Analysis of NG-108 Cells

**Figure 11:**
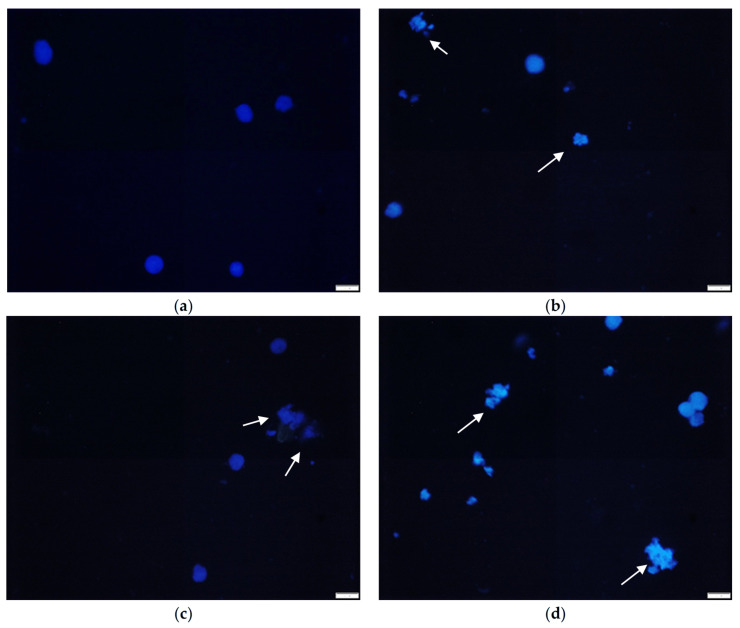
Representative fluorescence microscopy images of DAPI-stained NG-108 cells after 1 h of exposure to ZnO NPs. (**a**) Control, (**b**) 10 μg/mL, (**c**) 50 μg/mL, and (**d**) 100 μg/mL. White arrows indicate fragmented nuclei (Scale bar, 20 μm).

To investigate the mechanism of cell death induced by ZnO NPs, we performed DAPI staining to assess nuclear morphology in NG-108 cells after 1 h of exposure (Figure 11). Quantitative analysis revealed a dose-dependent increase in the percentage of cells exhibiting nuclear fragmentation, a hallmark of apoptosis (Table 3, Figure 11).

The percentage of cells with normal nuclei decreased significantly with increasing ZnO NPs concentration. At 10 μg/mL, approximately 25.40% of cells showed nuclear fragmentation, increasing to 36.40% at 50 μg/mL and 45.46% at 100 μg/mL. These results suggest that ZnO NPs induce apoptosis in NG-108 glioblastoma cells in a dose-dependent manner.

The observed nuclear fragmentation correlates well with the decreased cell viability measured by the MTT assay, supporting apoptosis as a primary mechanism of ZnO NPs -induced cell death in glioblastoma cells. This finding is consistent with previous studies suggesting that ZnO NPs can trigger apoptotic pathways through ROS generation and DNA damage [67,75,76,77].

### 3.12. Mechanistic Insights into Selective Cytotoxicity

The observed selectivity results from three convergent mechanisms operating under dark laboratory conditions. First, defect-mediated ROS generation occurs through oxygen vacancy defects (characteristic 550 nm photoluminescence emission) that generate reactive species via surface reactions: $V_{O}+H_{2}O\to V_{O}^{+}+{OH}^{-}+e^{-}$, followed by $O_{2}$ reduction to superoxide and hydrogen peroxide, explaining cytotoxic activity without external activation. Second, pH-responsive dissolution enabled by zeta potential characteristics (pHPZC = 7.46) promotes preferential zinc ion release in acidic tumor microenvironments (pH 5.5–6.5) versus physiological pH (7.4), while maintaining optimal dispersion stability (±2.5 mV) for cellular uptake. Third, differential cellular vulnerability manifests as rapid cytotoxicity in glioblastoma cells (1 h) versus delayed effects in retinal cells, reflecting enhanced nanoparticle uptake in cancer cells and their compromised antioxidant capacity compared to normal cells. This multi-factorial mechanism explains the 2.3-fold selectivity index, demonstrating intrinsic anticancer activity without external activation.

## 4. Discussion

The present study investigated the cytotoxic effects of biogenically synthesized ZnO NPs on primary retinal cells and NG-108 glioblastoma cells, aiming to evaluate their potential as a targeted therapy for glioblastoma. Our findings demonstrate a significant difference in sensitivity between normal neural cells and glioblastoma cells, suggesting a potential therapeutic window for ZnO NP-based treatments. The observed differential cytotoxicity is a key finding, with primary retinal cells showing resistance to ZnO NPs at lower concentrations (10 and 50 μg/mL) during short-term exposure (1 h), while glioblastoma cells exhibited significant cytotoxicity under the same conditions. This selective toxicity aligns with previous studies on other cancer cell types [78,79] and could be attributed to several factors, including differences in metabolic rates, cell membrane properties, and antioxidant capacities between normal and cancer cells [80].

Importantly, our study demonstrates that biogenic ZnO NPs achieve selective anticancer activity under standard dark laboratory conditions, eliminating the need for external photoactivation. The mechanistic foundation was elucidated through photoluminescence analysis revealing substantial oxygen vacancy defects (evidenced by intense 550 nm emission), which enable intrinsic ROS generation through defect-mediated surface reactions with cellular environments. This dark cytotoxicity, combined with pH-responsive dissolution behavior (pHPZC = 7.46), provides practical advantages over photodynamic approaches requiring controlled irradiation.

The time-dependent effects observed in primary retinal cells, where prolonged exposure resulted in increased cytotoxicity, suggest a cumulative impact of ZnO NPs on cellular function. This phenomenon could be explained by gradual nanoparticle uptake, zinc ion release from dissolving NPs, or the accumulation of cellular damage over time [66]. These findings emphasize the importance of considering exposure time when developing ZnO NP-based therapies and highlight the need for careful dosing strategies to minimize damage to healthy tissue [71].

Our nuclear morphology analysis of NG-108 cells exposed to ZnO NPs revealed a dose-dependent increase in nuclear fragmentation, a hallmark of apoptosis. This observation is consistent with previous studies suggesting that ZnO NPs induce apoptosis in various cancer cell types. The apoptotic mechanism involves defect-mediated ROS generation operating continuously under dark conditions, leading to sustained oxidative stress, DNA damage, and mitochondrial dysfunction. This intrinsic activity pattern differs from burst-type ROS generation in photodynamic therapy, potentially offering advantages in terms of sustained therapeutic pressure on cancer cells while allowing normal cells time for repair mechanisms. The predominance of apoptosis as the cell death mechanism is favorable for potential therapeutic applications, as it is less likely to induce inflammation compared to necrotic cell death [71,81,82].

The selective cytotoxicity of ZnO NPs towards glioblastoma cells observed in this study has significant implications for the development of novel glioblastoma therapies. The differential sensitivity between normal and cancer cells suggests that ZnO NPs could potentially target glioblastoma cells while sparing healthy neural tissue, addressing a major challenge in current glioblastoma treatments [83,84,85]. Furthermore, the unique mechanism of action of ZnO NPs, involving ROS generation and direct cellular damage, may help overcome the resistance mechanisms that limit the efficacy of conventional chemotherapies [71,86]. The nanoscale size of ZnO particles may also facilitate their passage through the blood–brain barrier, a significant obstacle in glioblastoma treatment. The dark cytotoxicity eliminates complications associated with light penetration in brain tissue, a significant advantage for glioblastoma treatment where deep tissue access is challenging.

Additionally, the apoptosis-inducing properties of ZnO NPs could potentially synergize with existing treatments, such as temozolomide or radiation therapy [87].

While our results are promising, several limitations should be addressed in future studies. The use of cell cultures, while informative, does not fully recapitulate the complexities of the tumor microenvironment or the blood–brain barrier. In vivo studies using appropriate animal models are necessary to validate these findings. Further investigation into the long-term effects of ZnO NPs on both normal and cancer cells is needed to assess their safety profile for potential clinical applications. More detailed mechanistic studies are required to fully understand the basis for the differential sensitivity between normal and cancer cells. The influence of nanoparticle size, shape, and surface modifications on their biological effects should be explored to optimize their therapeutic potential. Studies on the in vivo distribution, accumulation, and clearance of ZnO NPs are crucial for assessing their potential for clinical translation.

## 5. Conclusions

This study demonstrates the successful biogenic synthesis of ZnO NPs using *Bacillus licheniformis* strain TT14s, producing crystalline nanoparticles with a mean diameter of 19.37 ± 5.28 nm and a hexagonal wurtzite structure confirmed by XRD analysis. The synthesis protocol yielded high-purity ZnO with minimal organic residue after 350 °C calcination, as evidenced by the characteristic Zn-O stretching vibration at 381 cm^−1^ in FTIR analysis and lattice spacing of 0.244 nm corresponding to the (101) plane in HRTEM imaging.

The primary finding is the intrinsic selective cytotoxicity of ZnO NPs against NG-108 glioblastoma cells versus primary retinal cells under dark laboratory conditions. This selectivity is mechanistically attributed to: (1) defect-mediated ROS generation confirmed by characteristic 550 nm photoluminescence emission from oxygen vacancy defects, (2) pH-responsive dissolution behavior (pHPZC = 7.46) favoring zinc ion release in acidic tumor microenvironments, and (3) differential cellular uptake in rapidly proliferating cancer cells with compromised antioxidant systems. At 100 μg/mL, glioblastoma cell viability dropped to 36.07% within 1 h, while retinal cells maintained 78.9% viability under identical conditions, representing a practical 2.3-fold higher selectivity index.

DAPI nuclear staining quantitatively confirmed apoptotic cell death, with nuclear fragmentation increasing from 25.4% at 10 μg/mL to 45.46% at 100 μg/mL in glioblastoma cells. This dose-dependent response validates apoptosis as the primary death mechanism rather than necrosis. The 1 h exposure time required for significant cytotoxicity suggests rapid cellular uptake and immediate biological activity. The intrinsic cytotoxic activity under dark laboratory conditions eliminates the need for external activation, providing practical advantages for therapeutic applications.

ZnO NPs demonstrate promise as selective anti-glioma agents with intrinsic dark cytotoxicity through validated mechanisms: defect-mediated ROS generation, pH-responsive dissolution, and enhanced cancer cell uptake. The elimination of external activation requirements provides significant practical advantages for therapeutic development. The green synthesis approach using *B. licheniformis* offers a scalable, environmentally sustainable platform with superior biocompatibility compared to chemical methods, suitable for clinical translation as a dark-active therapeutic agent for glioblastoma treatment.

## Figures and Tables

**Table 1:** Dose- and time-dependent cytotoxicity of zinc oxide nanoparticles (ZnO NPs) in primary retinal cells: cell viability percentages (mean ± SEM) from n = 3–4 independent experiments with N = 3–4 replicates per condition. Values with letter (a) show no significant differences (Tukey, *p* > 0.05).

Concentration	10 µg/mL			50 µg/mL			100 µg/mL		
Time	Mean %	SEM	N	Mean %	SEM	N	Mean %	SEM	N
Control	100.00 ^a^	0.12	3	100.00 ^a^	3.52	4	100	0.74	4
1 h	96.53 ^a^	0.22	3	89.68 ^a^	1.7	4	78.9	0.94	4
6 h	74.7	1.57	3	58.21	1.8	4	48.31	3.73	4
12 h	79.39	2.64	3	62.76	0.09	4	46.61	2.34	4
24 h	72.36	1.09	3	54.77	2.24	4	43.48	2.34	4

**Table 2:** Effect of ZnO NPs on NG-108 glioblastoma cell viability after 1 h of exposure. Data are presented as mean percentage of viable cells ± SEM (n = 3 independent experiments).

Concentrations (µg/mL)	Zinc Oxide NPs Incubated for 1 h		
	Mean %	SEM	N
Control	100	1.22	3
10	70.41	4.29	3
50	58.23	3.15	3
100	36.07	1.89	3

**Table 3:** Percentage of NG-108 cells with normal nuclei after 1 h of exposure to ZnO NPs. Data are presented as mean percentage ± SEM (n = 3 independent experiments).

Zinc Oxide Concentration	Mean (%)	SEM	n
Control	100	0.441	3
10 µg/mL	74.6	1.076	3
50 µg/mL	63.6	1.417	3
100 µg/mL	54.54	0.782	3

## Data Availability

The authors verify that all data obtained in this study are presented in this published article.
